# Therapy of diarrhea in COVID-19 with external treatment of traditional Chinese medicine: A protocol for systematic review and meta-analysis

**DOI:** 10.1097/MD.0000000000024120

**Published:** 2021-01-08

**Authors:** Cheng Cheng, Yashuang Huang, Li Xie, Xinghui Zhu, Dongmei Chen, Cisong Cheng

**Affiliations:** School of Basic Medicine, Chengdu University of Traditional Chinese Medicine.

**Keywords:** COVID-19, diarrhea, meta-analysis, protocol, systematic review, traditional Chinese medicine

## Abstract

**Background:**

Evaluating the effectiveness and safety of external treatment of traditional Chinese medicine therapy for COVID-19 with diarrhea is the primary purpose of this systematic evaluation program.

**Methods:**

We will search the randomized controlled trials from inception to November 2020. The following database is our focus area: Cochrane Central Register of Controlled Trials, Embase, PubMed, Web of Science, China National Knowledge Infrastructure (CNKI), Chinese Biomedical Database (CBM), VIP database for Chinese technical Periodicals, and Wanfang Database. We will choose articles published both in Chinese and English. Two reviewers will conduct the study selection, data extraction, and assessment independently. The assessment of risk of bias and data synthesis will be carried out using Review Manager Software V.5.3.

**Results:**

The results will provide high-quality synthesis of current evidence for researchers in this subject area.

**Conclusion:**

This studys decision will provide evidence of whether external treatment of traditional Chinese medicine is an effective and safe intervention for coronavirus disease 2019 with diarrhea.

**Registration number:**

INPLASY2020110095 (DOI number: 10.37766/inplasy2020.11.0095).

## 1 Introduction

In December 2019, the COVID-19 outbreak caused by SARS-CoV-2 first appeared in Wuhan. The new coronavirus (SARS-CoV-2) is highly contagious and can infect people of all ages, mainly through respiratory droplets and close contact.^[1–3]^ The virus is spreading rapidly around the world. As of November 24, 2020, the World Health Organization (WHO) reported that in 220 countries/regions, there were more than 58.71 million confirmed cases and 1.38 million deaths. The number of confirmed cases and deaths is increasing every day. The COVID-19 pandemic poses a huge threat to global public health security. In the current pandemic, most attention is still only focused on the respiratory symptoms of the disease. However, it must be emphasized that the number of COVID-19 patients with gastrointestinal symptoms such as diarrhea is increasing.^[4–7]^ COVID-19-associated diarrhea occurrence has been reported at 2.0%,^[8]^ 3.0%,^[9]^ 7.3%,^[10]^ 10.1%,^[10]^ and 49.5%.^[11]^

Traditional Chinese medicine has accumulated thousands of years of experience in the prevention and treatment of infectious diseases. External treatment of Traditional Chinese medicine is an important part of Traditional Chinese medicine. External treatment of Traditional Chinese medicine refers to treatment methods that act on the body surface or from outside the body in addition to oral administration or injection, including acupuncture, moxibustion, acupoint application, massage, fire acupuncture, ear points, etc. It is simple operation, few adverse reactions, and quick clinical onset are widely used to treat diarrhea.

Acupuncture is an important part of the external treatment of Chinese medicine, which has its own distinctive features and unique advantages. Modern clinical and experimental studies have shown that acupuncture can regulate human immune function and has anti-inflammatory and anti-infection effects.^[12]^ It can improve the symptoms of the digestive tract.

Moxibustion, as one of the traditional Chinese medicine external treatment methods, uses burning mugwort leaves to stimulate acupuncture points or specific parts on the body surface to promote the bodys self-regulation function, thereby achieving the purpose of disease prevention and treatment. China has thousands of years of application history of moxibustion to prevent and treat diseases. Modern research shows that moxibustion can adjust the respiratory systems functions, immune system, and other systems, promote metabolism, and enhance immune function, especially in treating chronic diseases and preventive health care.^[13]^

Tuina is one of the commonly used clinical external treatment methods of traditional Chinese medicine. The therapy acts on specific meridian points on the human body surface through certain techniques to speed up venous blood and lymph fluid return and improve gastrointestinal function.

Acupoint application is a more common method of external treatment methods of traditional Chinese medicine. Acupoint applicator stimulates acupuncture points on the body surface, stimulates meridians, regulates blood, improves blood circulation, promotes and adjusts the bodys immune function, thereby exerting its effect on the digestive system and improving the symptoms of diarrhea. At present, there is still a lack of evidence-based medical evidence to support the treatment of patients with COVID-19 of diarrhea using external treatment of Traditional Chinese medicine. Therefore, it is necessary to conduct further review to provide evidence for clinicians.

## 2 Methods

### 2.1 Study registration

The systematic review protocol has been registered in the International Platform of Registered Systematic Review and Meta-analysis Protocols (INPLASY2020110095). The protocol refers to the guide book of Preferred Reporting Items for Systematic Reviews and Meta-Analyses Protocols (PRISMA-P).^[14]^

### 2.2 Inclusion criteria for study selection

#### 2.2.1 Type of study

We will include articles related to traditional Chinese medicine external treatment for treating COVID-19 patients with diarrhea. All included items were randomized controlled trials. Due to language limitations, we will choose article published both in Chinese and English as long as it meets our inclusion criteria. Non-RCTs, quasi-RCTs, series of case reports, animal trials, and laboratory studies will be excluded.

#### 2.2.2 Type of participant

Patients diagnosed with COVID-19 and symptoms of diarrhea will be included regardless of sex, age, race, education, and economic status.

#### 2.2.3 Type of intervention

Interventions will include the external treatment of traditional Chinese medicine. External treatment of traditional Chinese medicine will consist of acupuncture, massage, fire needle, moxibustion, etc. Other conventional Chinese therapies will be excluded. We will compare the following interventions: treatments other than traditional Chinese medicine (e.g., usual or standard care, placebo, wait-list controls).

#### 2.2.4 Type of outcome measure

##### 2.2.4.1 Primary outcomes

Frequency of diarrhea and fecal texture. Compare the number of daily defecation and fecal consistency before and after treatment.

##### 2.2.4.2 Secondary outcomes

Accompanying symptoms (such as myalgia, expectoration, stuffiness, runny nose, pharyngalgia, anhelation, chest distress, dyspnea, crackles, headache, nausea, vomiting, anorexia, diarrhea) disappear rate, negative COVID-19 results rate on 2 consecutive occasions (not on the same day), CT image improvement, average hospitalization time, the occurrence rate of common type to a severe form, clinical cure rate, and mortality.

##### 2.2.4.3 Additional outcomes

Adverse events.

### 2.3 Data sources

We will perform literature searches using the following electronic bibliographic databases from inception to November 2020: Cochrane Central Register of Controlled Trials, Embase, PubMed, Web of Science, China National Knowledge Infrastructure (CNKI), Chinese Biomedical Database (CBM), VIP database for Chinese technical Periodicals, and Wanfang Database.

### 2.4 Search strategy

The search terms on PubMed are as follows: acupuncture (e.g., “acupuncture” or “acupuncture therapy” or “body acupuncture” or “manual acupuncture” or “electroacupuncture” or “fire needling” or “plum blossom needling”); moxibustion (e.g., “moxibustion” or “moxibustion therapy”); massage (e.g., “acupoints” or “tuina” or “manipulation”); acupoint application; diarrhea; COVID-19 (e.g., “Corona Virus Disease 2019” or “Corona Virus” or “new coronavirus” or “2019 novel coronavirus”); RCT (e.g., “randomized” or “randomly” or “clinical trial”). Combinations of Medical Subject Headings (MeSH) and text words will be used. The search term in the Chinese database is the translation of the above word. The complete PubMed search strategy is summarized in Table 1.

**Table 1:** Search strategy for the PubMed database.

Number	Search items
1	Acupuncture
2	Acupuncture therapy
3	Body acupuncture
4	Manual acupuncture
5	Electro acupuncture
6	Fire needling
7	Plum blossom needling
8	1 or 2–7
9	Moxibustion
10	Moxibustion therapy
11	9 or 10
12	Massage
13	Acupoints
14	Tuina
15	Manipulation
16	12 or 13–15
17	Acupoint application
18	Diarrhea
19	COVID-19
20	Corona Virus Disease 2019
21	Corona Virus
22	New coronavirus
23	2019 novel coronavirus
24	19 or 20–23
25	Randomized controlled trial
26	Randomized
27	Randomly
28	Clinical trial
29	25 or 26-28
30	8 and 11 and 16 and 17 and 18 and 24 and 29

### 2.5 Data collection and analysis

#### 2.5.1 Selection of studies

**Figure 1:**
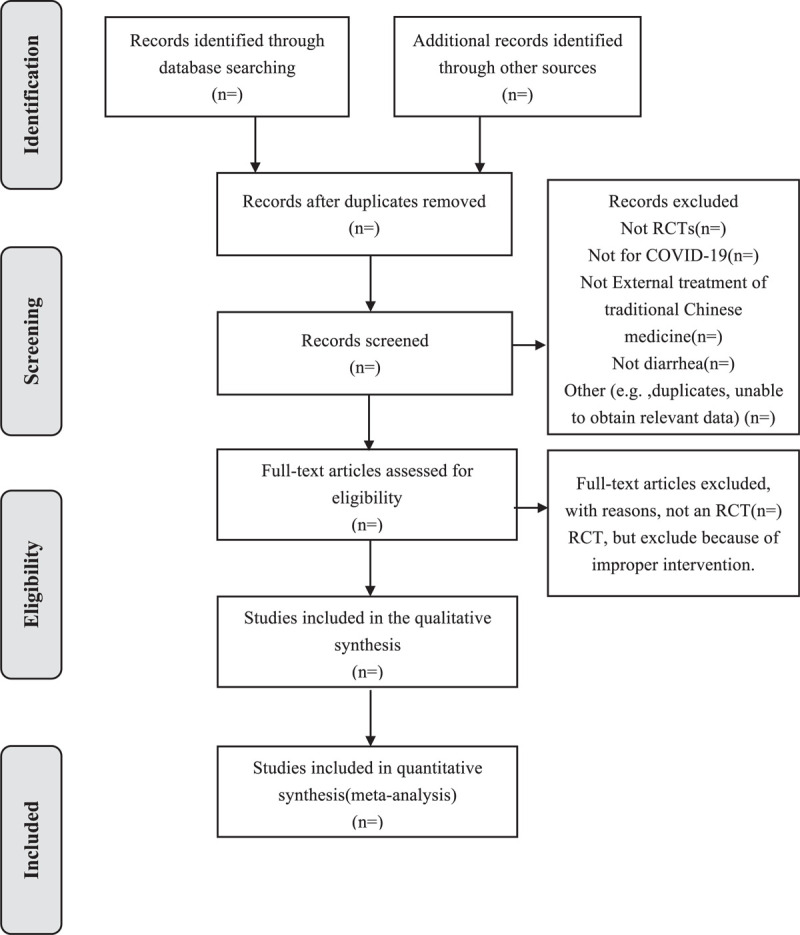
Flow chart of the study.

We will use EndNote X9 software to manage the records of searched electronic databases. Before searching the literature, all reviewers will discuss and determine the screening criteria. After the screening requirements are clearly defined, 2 authors will independently scan all the records from the title and abstract, and all irrelevant literature will be removed. Then, full manuscripts of all remaining studies will be further identified to check if they meet all inclusion criteria. If there is any disagreement between the 2 authors in the literature data extraction, a third article participant will discuss the decision together. The detail of the study selection will be presented in a PRISMA flow diagram (Fig. 1).

#### 2.5.2 Risk of bias analysis

We will evaluate all the included studies according to the Cochrane Handbook guidelines for Systematic Reviews of Interventions. Two researchers would independently assess the evaluation above. Any discrepancies will be resolved by consensus or by a discussion with a third reviewer. Evaluation items contain the following 7 items: random sequence generation, allocation concealment, outcome evaluators, blinding of participants and personnel, incomplete outcome data, selective reporting, and other bias sources. The studies risk of bias will be divided into 3 levels (low risk, high risk, and unclear).

#### 2.5.3 Dealing with missing data

If we identify any unclear or missing data, we will try every means to contact the articles corresponding author, including sending emails or making a phone call. If we are unable to obtain the missing data, the study will be excluded from the analysis.

### 2.6 Statistical analysis

#### 2.6.1 Data synthesis

We will use Review Manager 5.3 and STATA 15.1 software for meta-analysis. The *I*^2^ test will be used to detect heterogeneity between trials. When the *I*^2^ test value >50%, there is significant statistical heterogeneity, and we will use a random-effects model. When the *I*^2^ test value is less than or equal to 50% and no statistical heterogeneity is found, the fixed effects model is used for data synthesis. All participants will discuss possible causes from a clinical and methodological perspective and provide descriptive or subgroup analysis.

#### 2.6.2 Subgroup analysis

If possible, a subgroup analysis will be performed to explain the reasons for the heterogeneity. Factors such as different types of control interventions and different outcomes will be considered.

#### 2.6.3 Sensitivity analysis

Suppose there is apparent heterogeneity in the tests included after the subgroup analysis. In that case, we will conduct a sensitivity analysis based on sample size, research design, heterogeneous quality, methodological quality, and statistical model to exclude quality Defect tests to ensure the analysis results stability.

#### 2.6.4 Assessment of reporting biases

If the number of studies included in a specific outcome index is no less than 10, we will generate funnel plots (effect size against standard error) to investigate publication bias.

#### 2.6.5 Quality of evidence

This paper will use the evidence quality rating method to evaluate the results obtained from this analysis. The evidence quality will be ranked by 4 levels: high quality, moderate quality, low quality, and deficient quality.

#### 2.6.6 Ethics and dissemination

Since the data used in this systematic review will be collected from published studies, ethical approval is not required.

## Author contributions

**Conceptualization:** Cheng Cheng.

**Data curation:** Cheng Cheng, Li Xie.

**Funding acquisition:** Yashuang Huang.

**Methodology:** Cheng Cheng, Yashuang Huang.

**Project administration:** Cheng Cheng, Xinghui Zhu, Dongmei Chen.

**Resources:** Li Xie, Dongmei Chen.

**Software:** Xinghui Zhu.

**Writing – original draft:** Cheng Cheng.

**Writing – review & editing:** Cheng Cheng, Cisong Cheng.
